# Can clinical ethics committees be legitimate actors in bedside rationing?

**DOI:** 10.1186/s12910-019-0438-y

**Published:** 2019-12-19

**Authors:** Morten Magelssen, Kristine Bærøe

**Affiliations:** 1 Centre for Medical Ethics, Institute of Health and Society, University of Oslo, Oslo, Norway; 2 Department of Global Public Health and Primary Care, University of Bergen, Kalfarveien 31, N-5018 Bergen, Norway

**Keywords:** Bedside rationing, Clinical ethics committees, Legitimacy, Priority setting, Resource allocation

## Abstract

**Background:**

Rationing and allocation decisions at the clinical level – bedside rationing – entail complex dilemmas that clinicians and managers often find difficult to handle. There is a lack of mechanisms and aids for promoting fair decisions, especially in hard cases. Reports indicate that clinical ethics committees (CECs) sometimes handle cases that involve bedside rationing dilemmas. Can CECs have a legitimate role to play in bedside rationing?

**Main text:**

Aided by two frameworks for legitimate priority setting, we discuss how CECs can contribute to enhanced epistemic, procedural and political legitimacy in bedside rationing decisions. Drawing on previous work we present brief case vignettes and outline several potential roles that CECs may play, and then discuss whether these might contribute to rationing decisions becoming legitimate. In the process, key prerequisites for such legitimacy are identified. Legitimacy places demands on aspects such as the CEC’s deliberation process, the involvement of stakeholders, transparency of process, the opportunity to appeal decisions, and the competence of CEC members. On these conditions, CECs can help strengthen the legitimacy of some of the rationing decisions clinicians and managers have to make.

**Conclusions:**

On specified conditions, CECs can have a well-justified advisory role to play in order to enhance the legitimacy of bedside rationing decisions.

## Background

### Bedside rationing and clinical ethics committees

In publicly funded health services there is a need to ration healthcare as claims on healthcare exceed available resources. Many countries have set up systems and institutions, such as the UK’s NICE, to promote fair priority setting. Less work has been done on bedside rationing – the practice of making rationing and allocation decisions close to patients, typically by practicing clinicians themselves. Important dilemmas arise in bedside rationing; however, in practice, clinicians might not be *aware of priority setting principles or mechanisms* for promoting fair bedside rationing, and might *lack support* from managers, guidelines, legislation, and politicians for the difficult rationing decisions that sometimes have to be made [1].

Clinical ethics committees (CECs) have been proposed to be of some value in mitigating both these deficiencies [2]. CECs are multidisciplinary committees tasked with increasing clinician awareness of ethical problems and solutions in their workday and with providing deliberations and advice on difficult clinical-ethical problems. CECs have long been features of many hospitals in the Western world. Recently it has been argued that CECs might also play several constructive roles in issues concerning priority setting, resource use and bedside rationing [2].

### Legitimacy in rationing: two theoretical frameworks

However, problems of bedside rationing often have a special feature that distinguish them from other clinical-ethical problems. As bedside rationing issues concern scarce resources, the outcome is of relevance to further stakeholders than the actors directly involved, especially when the decision requires resources that could have been spent differently. For a resource driving outcome to be legitimate, therefore, more than the assent of the clinicians and patient directly involved is required. There is then a question whether CEC deliberations about issues of bedside rationing can contribute to the *legitimacy* of processes conducted and conclusions reached. Thus we ask: What does it take for the CEC to be a legitimate actor in bedside rationing dilemmas in the hospital?

Limit-setting decisions in health carried out on a population level can be subjected to regulations by specific criteria for just allocation of health care. However, as pointed out by Daniels and Sabin [3], we cannot expect people to agree on what specific criteria or principles should be applied to promote justice in these decisions. Rather, we must ensure that the way the criteria or principles are reached – the decision-making processes – are such that people perceive them as fair and legitimate. The procedural approach that Daniels and Sabin advocate, ‘Accountability for Reasonableness’ (A4R), stresses the importance of transparency and accountability. This framework consists of four procedural conditions that are necessary for the outcome of priority setting processes to be legitimate: “*transparency about the grounds for decisions; appeals to rationales that all can accept as relevant in meeting health care needs fairly; and procedures for revising decisions in light of challenges to them*”; and in addition, there must be institutions in place to enforce the previous three requirements [3].

When subjected to this kind of regulating process, the decisions that are the outcomes of the process can be justified as legitimate. However, the kind of rationing decisions Daniels and Sabin have in mind are those concerning patient groups, not individuals.

Clinical priority setting decisions about individual claims on healthcare (‘bedside’) can also be perceived as *indirectly* subject to similar conditions of procedural justice, as clinicians are expected to comply with evidence-based clinical guidelines when there is no obvious reason to consider them irrelevant for the case at hand. The development of evidence based clinical guidelines inevitably relies upon normative considerations [4–7] which can – and should – also be subjected to accountability. Norheim has developed a framework wherein the acceptability of processes of development of guidelines are evaluated, and where guidelines themselves are considered as tools for priority setting [8, 9].

Evidently, clinical guidelines cannot provide conclusive guidance on every clinical decision that has to be made. For instance, while emergency department triage is guided by principles, decisions ultimately rely on discretionary considerations of how these principles apply to the particular cases at hand. Sometimes there might be reasons to *deviate* from guidelines by providing or limiting care and the choice whether to do so constitutes a dilemma. At other times there are conflicting views as to whether existing guidelines apply or not. In such situations, what can make decisions legitimate?

To shed light on this question, Bærøe developed a framework to complement the A4R framework [10]. This approach was inspired by the identified lack of transferability of legitimacy from macro-level decisions about claims of patient groups to micro-level claims of individuals [11]; the contextual conditions for making ethical judgments do not coincide. Based on analyses of the concept of a ‘healthcare claim’ and of clinicians’ scope of responsibility respectively, as well as an appreciation of the theoretical grounding of A4R, the framework details conditions for legitimacy for clinical decisions with priority-setting consequences at the micro (bedside) level. Together, the A4R and Bærøe’s approach can be seen as mutually supporting accountable and legitimate decisions throughout a publicly funded healthcare system.^1^

### A role for CECs in bedside rationing?

Against this backdrop of two theoretical approaches to what makes rationing decisions legitimate we will explore the role of CECs in such decisions. We focus on situations of choice that have implications for priority setting/resource allocation and rationing and where the decision-makers are healthcare personnel (or managers) with the discretion to make judgments about bedside rationing. We make one crucial assumption: CECs do not have the mandate to *make* bedside rationing decisions, only to assist in decision-making. There can be different reasons for this. For example, it is not practical that others than those who are responsible for the overall care are delegated this responsibility, and CECs have not originally possessed any particular competence that would make them better suited to carry the political mandate of distributing health care. The question is then to what extent these committees nevertheless have the potential to influence the overall legitimacy of these decisions.

After setting out the seven criteria for legitimacy in Bærøe’s framework we present two empirical, real world priority setting dilemmas wherein CECs were involved, outlining six roles CECs played in the two cases and might play in priority setting cases in general. In order to reach general conclusions with relevance across particular settings, we then discuss the legitimacy of these roles rather than the particular cases in question. For these roles to be considered legitimate in lack of any particular, formal decision-making authority, they will have to be assessed according to requirements addressing the broader picture of both macro- and micro-level requirements for fair priority setting in general. Bærøe’s framework is a response to exactly this complex, general challenge as it addresses the overall legitimacy of the implicit priority setting taking place on the micro-level under influences of macro-level decisions. Therefore, when being without any formally assigned authority, the roles of CECs in contributing to bedside rationing will have to be subjected to at a minimum the same requirements of legitimacy as clinician/managerial bedside decisions. With Bærøe’s normative framework as the backdrop we then discuss whether and how CECs by fulfilling the six roles contribute to the legitimacy of the decisions reached. Leaning up on Ives and colleagues’ recently published consensus-based standards of practice for empirical bioethics [12], we acknowledge the requirement for unpacking and openly exposing how the integration of empirical findings and normative arguments takes place within this piece of empirical bioethics work. Also, we will clarify the fundamental epistemic assumption for this approach.

Based on a diversity of empirical cases, Magelssen et al. identified several roles that CECs play in bedside rationing under the normative aim of considering their practical, productive contribution. However, such ‘positive’ roles might not be justified as ‘legitimate’. In order to make further normative judgements about ‘legitimacy’, the relevance of these roles is therefore assessed according to a normatively more broadly construed ethico-political framework of ‘legitimacy’. By doing this, we assume a constructivist approach to what tasks CECs can be justified or not in carrying out; our understanding of the ‘should and should not’ is socially constructed. Moreover, when translating theoretical work into a field of practice, we assume the epistemic requirement of establishing empirical knowledge to allow sound conclusions [13].

## Main text

### Reasonable clinical judgments and aspects of legitimacy

Bærøe’s framework for reasonable clinical judgments with priority-setting consequences consists of six requirements on clinicians and one requirement on the self-regulation of the medical profession, requirements that will now be explained briefly. The starting-point is the recognition that clinicians have dual responsibilities; towards the patient in front of them and towards the entire patient population of the health care system (which shares the same public funding), respectively, coupled with the call to ensure equal treatment across individuals and groups (horizontal equity), as well as justified unequal treatment among individuals and groups (vertical equity). This leads to four different aims of justice that surround clinical decision-making in health:

- *horizontal equity* demanding equal treatment of cases considered equal within the clinician’s own patient population
- *vertical equity* regarding discrimination between the needs of the clinician’s own patients
- *contributing to vertical equity* by unequal treatment of unequal cases within the whole patient population of a healthcare system
- *contributing to horizontal equity* by equal treatment of equal cases within the whole patient population of a healthcare system.

Reflection on these four aims of justice led to the framework consisting of seven requirements summarized in Table 1. The framework can appear daunting, but the gist is not that each clinical decision must conform fully to all requirements in order to be legitimate; rather, the requirements together form an ideal to strive towards and are helpful in showing how the legitimacy of decisions might be increased.

**Table 1:** Seven requirements for legitimacy of clinical decisions with priority-setting consequences. From Bærøe [10]

	Requirement	Explanation (conditions supported in parentheses)
1	Self-reflection	Explicit reflection on applicable goals of healthcare and principles for distribution (supports condition (a))
2	Search for all relevant arguments	Identification of context- and patient-related reasons to justify deviation from guideline (b)
3	Impartiality	Recognition of impartiality (a-b)
4	Political consequences	Recognition of the political consequences of the claims put forward (c)
5	Prioritised services	A stable perception/justification of what kind of services the healthcare service should prioritise (c)
6	Reasonable justification	Justification of claims on healthcare so that they would be acceptable to colleagues sharing this aim of justification (d)
7	Professional self-regulation	Institutionalisation of requirements 1–6 supports all four conditions (a-d) and makes the performers accountable towards health authorities and stakeholders

These requirements reflect a conceptualization of ‘legitimacy’ in terms of both *procedural* and *epistemic* dimensions. Requirement 7 underscores the procedural aspect of the legitimacy of the judgments made by clinicians in individual cases; if these conditions are in place, these decisions can be approved as legitimate. Requirements 1–6 all work towards epistemological steering of the outcome of the judgement towards reasonableness by specifying what to justify and demands on the justification. Requirement 6 explicitly demands a fair-minded approach for justification. Requirement 3 introduces the formal claim on impartiality, and reflects that recognition of the moral equality of stakeholders should structure the reasoning. Requirements 1–2 and 4–5 all imply formal issues considered relevant to apply when promoting fair-minded judgements about bedside rationing.

When these requirements are considered in a broader societal and political context, it becomes clear that the legitimacy of the normative basis clinicians derive their justified claims on healthcare on, is not an isolated professional concern. Health authorities in a publicly funded system cannot accept a clinical practice based on whatever clinicians would agree among themselves to consider fair, unless this normative basis also resonates well with general, accepted political principles for distribution within society at large. To ensure this to happen requires taking into account input from other stakeholders than the clinicians themselves. This brings in a broader, *political* dimension to the conceptualisation of ‘legitimacy’ regarding bedside rationing. Stakeholders, e.g. patients, proxies, other healthcare personnel and tax-paying citizens in general, must also be allowed to influence the common normative basis for deriving prioritized and rationed health care claims. As a prerequisite, the normative principles justifying individual limit-settings will have to be publicly accessible and transparent, and opportunities for appeal and revisions made available for anyone interested in doing so.

### CECs’ involvement in bedside rationing

Any justified, legitimate role of CECs involved in bedside rationing – considered as a procedural condition itself – can now be assessed according to whether and how these committees manage to strengthen the procedural, epistemic and political dimensions of legitimacy produced by the decision-making processes. A recently published paper presents an overview of potential roles CECs can take on in bedside rationing, based on real word examples [2]. This provides us with a very useful starting point to identify how CECs actually *can* influence bedside rationing/resource allocation. We are interested in how these committees can do so in a normatively justified manner. Therefore, we will discuss how these approaches contribute to legitimacy in line with the requirements for reasonable clinical priority- and limit setting and the additive, political requirement that stakeholders are given the opportunity to influence the normative basis for these decisions.

An analysis of 38 Norwegian CECs’ work with ethical problems of priority setting and resource use identified six roles that CECs can play in handling such cases (Table 2) [2]. We illustrate the roles with two cases drawn from the Norwegian study.

**Table 2:** Roles and possible impact of CECs dealing with priority issues (adapted from Magelssen et al.) [2]

Role	Potential impact
Analyst	Clarify values/principles at stake and the impact of decisions
Advisor	Solve concrete dilemmas
Moderator	Contribute to fairer decision-making processes
Disseminator	Create awareness and disseminate knowledge among clinicians
Coordinator	Connect different levels of healthcare organization
Guardian of values and laws	Ensure legitimacy and fairness in line with common values

We now present two CEC cases concerning bedside rationing, taken from Magelssen et al. [2] and summarized and adapted by the present authors.

### Case 1

The hospital CEC was called on to give advice on the care of a patient who received continuous ventilator treatment in the home. A large care team was required, and municipal authorities wanted the patient to move to a nursing home to reduce costs and improve continuity of staffing – against the patient’s own preference. A team of CEC members went to the patient’s house to discuss and note the opinions of the patient and spouse. Next, the CEC held a meeting where other stakeholders, including municipal authorities and carers and hospital staff, came together to discuss what was perceived as a conflict between patient autonomy and fair resource use and justice. The CEC organised both the deliberations and the written consultation report by a structured deliberation method. In the report, the CEC detailed the information that had been presented to the committee, and discussed the trade-off between values and principles, the role of national priority setting criteria, and legal aspects. The committee’s advice to the municipality was to offer future care in the nursing home only.

### Case 2

A new drug had shown some effect for patients with metastatic lung cancer. Yet cost-effectiveness was very low with high drug costs and only modest effects. The national decision-making body had not yet concluded whether the drug should be covered by public funds and offered in public hospitals. The CEC was contacted by a physician as some patients now wanted to buy the drug themselves and have it administered by the hospital; how was the physician to respond? In Norway’s publicly funded healthcare system this practice would be unprecedented. There were no private clinics in the city that would offer to administer the treatment. If the hospital were to provide the treatment, then this would entail substantial additional use of resources such as outpatient visits, clinician time and attention and additional radiological examinations and blood tests. The CEC consulted several stakeholders and concluded that the principle of equal access, fundamental to Norwegian healthcare policy, should take precedence, and that it was therefore unacceptable that only those who were able to pay should receive the drug in a public hospital. Although the clinician was thankful for the CEC’s support and clear arguments, patients were dissatisfied and protests reached the media. The CEC’s proposed policy was later adopted hospital-wide by the management. The CEC also brought the dilemma to a national level, to be discussed in the National council for priority setting. A 2016 government white paper on priority setting outlined a similar policy, which was then adopted in parliament: when the evaluation of public coverage for new drugs is underway, the drugs will not be administered in public hospitals at the patient’s expense in the meantime.

### CECs as analysts

In the two cases the CECs acted as *analysts*. The CECs discussed the cases together with the different stakeholders, applying, explaining and clarifying relevant moral principles in the process. As an analyst, the CEC can take on the role as a dialogue partner and assist decision-makers in structuring their reflection towards reasonableness according to all requirements 1–6 in Bærøe’s framework. This presupposes that CEC members collectively possess the relevant competence in priority setting criteria and connected moral arguments and political theory to guide accordingly. In our experience, clinicians often possess only rudimentary knowledge in this area. However, many CECs employ an ethicist or philosopher who could perhaps assume special responsibility for this body of knowledge. For CECs to legitimately take on the role as analyst according to the framework, some measure of this kind of competence is certainly required. At the same time, the fact that CECs consist of several members that can contribute to enlighten more aspects of the issues than an individual clinician may manage on their own is likely to strengthen the epistemic dimension of reasonable justification of the decision according to requirements 2–6. It might matter here whether the CEC handles the case as a full committee or whether only some CEC members are involved in the consult. As for the political dimension of involvement of stakeholders underscored by the framework, this dimension is not strengthened by the CEC contributing as an analyst.

### CECs as advisors

A role as *advisor* implies the ability of CECs to reach a conclusion about reasonable bedside rationing. In the two cases, applying and weighing the morally relevant factors led the CECs to propose certain conclusions as the ethically most appropriate courses of action. The ability to give sound, justified advice relates to requirement 1–6 of Bærøe’s framework and hinges on the same skills highlighted above that enable the CEC to analyse the case; if not present, an advisory role cannot be legitimate. However, even though we may assume that the total competence of a CEC transcends the relevant competence of a clinician in illuminating a case, another issue occurs. Granting CECs an advisory role based on the privileged position of bringing in both disciplinary competence and multiple individuals’ perspectives might potentially be counter-productive. It might undermine the individual, epistemic project of the authorised decision-maker, the clinician, to reach fair conclusions. It is crucial that clinicians themselves – who are carrying the responsibility of the decisions – aim to develop adequate reasoning to apply consistently across cases. Reasoning *together with* the committee can certainly be a useful part of such an endeavour, but leaving it to the committee to produce advice (rather than help the clinician reach a conclusion) seems to create a mismatch between authorised discretion and responsibility. However, we find it likely that participation in CEC deliberations can provide clinicians with knowledge and a vocabulary for priority setting, thus equipping them to make future bedside rationing decisions on a sounder and more independent basis than before. Again, the advisor role of CECs alone does not support the political dimension of legitimacy.

### CECs as moderators

In both the two cases the CECs acted as *moderators* by structuring the deliberation process to give a hearing to arguments from the involved stakeholders. In case 1, different healthcare services (here, municipal and hospital, respectively) were brought together. In line with Bærøe’s framework, CECs are expected to organise processes that promote fair decision-making when acting as moderators. An often-invoked philosophical underpinning for CECs is discourse ethics, with its demands that all relevant stakeholders participate in deliberations held on equal terms, and that the best arguments rationally evaluated should prevail [14, 15]. These requirements resonate well with the framework’s demands on 2) searching for all relevant arguments, 3) impartiality, and 6) providing reasonable justification for entitled claims on care. Furthermore, CECs promote transparency, as stressed by the framework, by producing detailed case reports where conclusions are justified by arguments. However, there are further, more specific process requirements for priority setting decisions to promote fairness and legitimacy. The first is that there are more stakeholders than those that are directly involved in the case and likely to be present in the CEC meeting (i.e. patients, next of kin, healthcare personnel and managers), namely any other adversely affected patients, and other patients who could benefit from the opportunity costs. To promote impartiality (requirement 3 in the framework), the CEC should take the perspectives of any stakeholders not present into account in the deliberations. In the role of moderator the CEC is facilitating processes. For this to be a legitimate role, they must also possess knowledge and ability to translate relevant theoretical and practical insights on procedural fairness onto fuzzy, real-world conditions. This would for example not only require that stakeholders’ concerns are voiced, they will also have to be listened to and their concerns addressed in ways that give stakeholders reason to consider the conditions for the process fair [16]. In this respect case 1 appears to showcase an asymmetry in the CEC’s treatment of the patient and spouse on one hand and health professionals and managers on the other. Did the patient and spouse get to participate in the moral dialogue proper, or did they only get to voice their concerns? Were they allowed to contribute to and challenge the description of the values at stake and the definition of the ethical problem? Finally, according to the fair, procedural conditions in the A4R framework of Daniels and Sabin, it is crucial that stakeholders can make appeals about the criteria that justify priority setting, and arguably also when implemented in individual decisions. Again, to support impartiality (and thus legitimacy), CECs would have to be open to deliberate about potential appeals from stakeholders in the aftermath of consultations and assist in directing claims to the correct formal body.

### CECs as disseminators

In case 1 in particular, the CEC also acted as a *disseminator* in contributing to raised awareness and knowledge of priority setting principles and moral arguments. CECs act as disseminators when they make troublesome issues known to stakeholders and the public and when they inform stakeholders of institutionalised values, legal regulations and cultural, normative concerns to take into account (including the requirements set out above). In doing this, CECs disseminate knowledge that – when being correct and conveyed in ways that do not create misunderstandings – can only help support legitimacy along the three dimensions. For many CECs, in addition to case consultation an important function is to organize topical seminars and otherwise educate clinicians about issues in clinical ethics [17]. Raising issues of bedside rationing for discussion and informing about priority setting criteria and ethical principles can be a valuable way of creating awareness and encouraging clinician self-reflection and discussions within the professions (as per criteria 1 and 7 of Bærøe’s framework respectively).

### CECs as coordinators

CECs can also support legitimacy by influencing the political agenda by acting as *coordinators*, passing on principled issues from the clinical level. Case 2 illustrates the necessary interdependence between micro-, meso- and macro-level priority setting decisions. In case 2, the CEC raised the issue to the hospital management level, resulting in a hospital-wide policy which was in line with the CEC’s advice. Later the CEC also ensured that the issue received attention at the national level. A principled worry here, however, is whether different CECs would deem cases similarly as to whether the issues raised should be brought to higher levels of the organization or the political level. If it is left to the arbitrary judgments of CEC members (and their values and interests) when to do this or not, this does not support justified vertical equity on a population level (requirements 4–5) in the framework) and certainly not the procedural and political dimension of legitimacy. More precisely, legitimacy would ideally require transparent rules for when *not* to lift issues up for principled discussion. This would be to avoid that issues others want to have discussed on a principled level are stopped by the judgments of the members of the committee.

### CECs as guardian of values and laws

Finally, the CEC can act as a *guardian of values and laws*, in pointing out accepted social values and their impact on the case at hand. In case 2, the social value of equality of access was argued by the CEC to be particularly relevant. CECs have to impose self-regulation (according to requirement 7 of the framework) in order to be accountable towards health authorities and stakeholders. This involves critically assessing and justifying the values they purport to promote. In so far as CECs at the same time also strive for impartiality in addressing and scrutinizing stakeholders’ personal values – to also critically test the established regulations and expectations – this enhances all dimensions of legitimacy. If, on the other hand, CECs insist on narrowly understanding new clinical issues in light of the values previous cases have been appreciated according to, this undermines the same dimensions of legitimacy.

### Prerequisites for contributing to legitimacy

The discussion above has shown that CECs are able to contribute to procedural, epistemic and/or political legitimacy to the extent that they fulfil the six roles that CECs can play in issues of priority setting. However, the discussion has also highlighted prerequisites for CECs being able to fulfil these roles. These prerequisites may be grouped into three main categories: 1) the ability to adequately represent all stakeholders; 2) the ability to deliberate – and critically revise this deliberation on appeals – in a systematic manner that promotes impartiality and reasonableness, drawing on relevant clinical, ethical and legal arguments; and 3) transparency with respect to the deliberation and conclusions.

The extent to which CECs will be able to fulfil the prerequisites and thus contribute to legitimacy in practice is an interesting question which should be investigated empirically. The seven requirements for legitimacy of clinical decisions with priority-setting consequences and the requirements set out in the discussion of the six roles for the CEC could form the basis of evaluation criteria for CEC consultations on the topic.

## Conclusions

On the basis of criteria for legitimacy of clinical decisions with priority-setting consequences and roles that CECs might play in case consultations involving priority setting issues, we have shown that CECs have the potential to increase the legitimacy of such decisions. However, there are important prerequisites that must be in place for the CEC to contribute to legitimacy.

It seems likely that a CEC consultation will serve to *increase* the overall legitimacy of the decision above what single clinicians would achieve on their own even though not all prerequisites are fully in place. However, there is a danger that a CEC consultation that is flawed in respect of the prerequisites can confer a misleading ‘stamp of approval’ on the deliberation process and moral analysis. There is then a risk of bias and unjustified solutions and decisions based on fallacious reasoning or inadequate processes [18]. Involvement of the CEC might then serve to *decrease* legitimacy of decisions made. This is why close attention to the workings of CECs when handling issues of bedside rationing is so crucial.

## Data Availability

All data are presented in the article.

## Abbreviations

A4R: Accountability for reasonableness
CEC: Clinical ethics committee

## Footnotes

1 The roles of CECs which we are about to discuss can of course be relevant in a private payer or private insurance system also. However, we centre our discussion around publicly funded systems where calls for bedside rationing are likely to be more frequent.
